# The association of polymorphisms in nucleotide excision repair genes with ovarian cancer susceptibility

**DOI:** 10.1042/BSR20180114

**Published:** 2018-06-21

**Authors:** Zhiguang Zhao, Anqi Zhang, Yuan Zhao, Junmiao Xiang, Danyang Yu, Zongwen Liang, Chaoyi Xu, Qiong Zhang, Jianmin Li, Ping Duan

**Affiliations:** 1 Department of Obstetrics and Gynecology, The Second Affiliated Hospital and Yuying Children’s Hospital of Wenzhou Medical University, Wenzhou, Zhejiang 325027, China; 2 Department of Pathology, The First Affiliated Hospital of Wenzhou Medical University, Wenzhou, Zhejiang 325003, China

**Keywords:** case-control study, genetic susceptibility, nucleotide excision repair, ovarian cancer, polymorphism

## Abstract

Nucleotide excision repair (NER), the core mechanism of DNA repair pathway, was commonly used to maintain genomic stability and prevent tumorigenesis. Previous investigations have demonstrated that single nucleotide polymorphisms (SNPs) of NER pathway genes were associated with various types of cancer. However, there was no research elucidating the genetic association of entire NER pathway with ovarian cancer susceptibility. Therefore, we conducted genotyping for 17 SNPs of six NER core genes (*XPA, XPC, XPG, ERCC1, ERCC2*, and *ERCC4*) in 89 ovarian cancer cases and 356 cancer-free controls. Odds ratios (ORs) and 95% confidence intervals (CIs) were used to describe the strength of association. The result showed that both *ERCC1* rs11615 and *XPC* rs2228000 were significantly associated with reduced risk of ovarian cancer under dominant genetic model (adjusted OR = 0.35, 95% CI = 0.20–0.61, *P*=0.0002 and adjusted OR = 0.49, 95% CI = 0.30–0.81, *P*=0.005 respectively). In addition, *XPC* rs2228001 and *ERCC2* rs238406 had statistically significant association with the increased risk of ovarian cancer under dominant genetic model (adjusted OR = 1.72, 95% CI = 1.02–2.92, *P*=0.043 and adjusted OR = 2.07, 95% CI = 1.07–4.01, *P*=0.032 respectively). *ERCC1* rs3212986 were related with the increased risk of ovarian cancer under recessive model (adjusted OR = 2.40, 95% CI = 1.30–4.44, *P*=0.005). In conclusion, our results indicated that *ERCC1, XPC* and *ERCC2* might influence ovarian cancer susceptibility. Further research with large sample size is warranted to validate the reliability and accuracy of our results.

## Introduction

Ovarian cancer is the most deadly gynecological malignancy that mainly affects women in the period of childbearing age, perimenopause and postmenopause, accounting for 3% of newly diagnosed cancers among females in 2012 [1]. According to statistics, at least 75% of patients are diagnosed with advanced stage disease for the lack of early biomarkers for detection, effective chemoprevention, and asymptomatic characteristics [2,3]. The current treatment program for ovarian cancer mainly includes aggressive surgical approach and numerous chemotherapeutic agents [4]. However, 5-year relative survival of ovarian cancer remains in an extremely poor rate approximately 50% [5]. Therefore, there is an urgent need of some epidemiological and biological predictors for ovarian cancer in early stage.

DNA damage is involved in cancer and aging, and DNA repair plays an important role in the prevention of DNA from many deleterious effects, such as ultraviolet (UV) light, chemotherapeutic agents, and radiation [6,7]. Nucleotide excision repair (NER), the major mechanism in the process of DNA repair, was mainly implicated in the replacement of bulky, helix-distorting adducts with newly synthesized DNA segment [8,9]. Several researches have demonstrated the association between single nucleotide polymorphisms (SNPs) in NER pathway genes on many cancers susceptibility [10–12]. Hence, genetic alteration of NER-related genes may be closely related to the occurrence and development of ovarian cancer.

In the NER multistep reaction, it was mainly divided into three stages: DNA lesion recognition, incision and excision of double-stranded DNA, and gap-filling with DNA synthesis [13,14]. Xeroderma pigmentosum complementation group C (XPC)/human homolog B of Rad23 (HHR23B) complex is the initial recognizing protein responsible for the recruitment of relevant repair apparatus to the DNA lesion [15,16]. Besides, xeroderma pigmentosum complementation group A (XPA) also shows its high affinity with damaged DNA [6]. Excision repair cross-complementation group 2 (ERCC2) protein serves as a helicase subunit of transcription factor II H (TFIIH), which is known for its role in local unwinding of damaged strand and transcription initiation of RNA polymerase II [17,18]. During the period of double-strand breaks, the repair endonucleases excision repair cross-complementation group 1 (ERCC1)/excision repair cross-complementation group 4 (ERCC4) complex and xeroderma pigmentosum complementation group G (XPG) are responsible for cutting the damage-containing oligonucleotide [19,20]. These core proteins involved in the NER reaction play crucial role in the inhibition of tumorigenesis. In the present study, we performed a hypothesis-based association to explore the impact of SNPs in these core genes (*XPA, XPC, XPG, ERCC1, ERCC2*, and *ERCC4*) on the risk of ovarian cancer by genotyping a pool of 17 SNPs in 89 patients and 356 controls.

## Materials and methods

### Patients and controls

In the present case–control study, 89 ovarian cancer patients were enrolled by The Second Affiliated Hospital and Yuying Children’s Hospital of Wenzhou Medical University (WMU) from February 2007 to February 2017. All cases with ovarian cancer histology were confirmed by two gynecologic pathologists. The control group of 356 cancer-free women was also recruited from The Second Affiliated Hospital and Yuying Children’s Hospital of WMU in routine physical examination. All participants were frequency-matched to cases on age (±5 years) and race/ethnicity, and the people who had been diagnosed with malignant neoplasm or a family history of cancers were excluded in our research. All people included in the present research had signed a written informed consent. The research was approved by The Second Affiliated Hospital and Yuying Children’s Hospital of WMU.

### SNP selection and genotyping

The SNPs (Supplementary Table S1) of NER pathway genes were selected from the NCBI dbSNP database (http://www.ncbi.nlm.nih.gov/projects/SNP) according to the criteria described previously [12], and are potential function using SNPinfo online server (http://snpinfo.niehs.nih.gov/snpfunc.htm).

The TIANquick FFPE DNA Kit (Qiagen Inc., Valencia, CA) was applied to extract DNA genomic of all patients from paraffin-embedded tissue, while genomic DNA of the controls was extracted from the peripheral blood specimens using the TIANamp Blood DNA Kit (TianGen Biotech Co. Ltd.). A UV absorption spectrophotometer was used to detect DNA purity and concentration (Nano Drop Technologies Inc., Wilmington, DE).

Genotyping analysis was performed by real-time PCR with Taqman PCR master mix and ABI Prism 7900HT genetic detection system. In addition, approximately 5% samples were randomly selected as positive controls and negative controls for assessing the accuracy of genotyping results.

### Statistical analysis

The heterogeneity of the genotypes and ages between patients and controls was evaluated by Pearson’s *χ*^2^ test. The association between SNP and ovarian cancer risk were assessed by a generalized linear regression model, calculated as crude and adjusted odds ratios (ORs) and 95% confidence intervals (CIs). Deviation from Hardy–Weinberg equilibrium (HWE) among the controls group was assessed by a Chi-square test. All statistical tests were carried out by SAS software (Version 9.4; SAS Institute, Cary, NC, U.S.A.), with a two-sided *P*-value < 0.05 considering significant.

## Results

In the present study, we enrolled 89 ovarian cancer patients with an average age of 48.55 ± 11.66 months and 356 cancer-free controls with an average age of 45.37 ± 10.77 months. There was no significant difference between both groups (data not shown). The genotype frequencies of included SNPs among the controls conformed to HWE. As is shown in Table 1, our results demonstrated that *ERCC1* rs11615 was associated with a decreased risk of ovarian cancer with adjustment for age under dominant genetic model (adjusted OR = 0.35, 95% CI = 0.20–0.61, *P*=0.0002). Besides, compared with the carriers of *ERCC1* rs3212986 AC/CC genotype, the carriers of AA genotype had a significant association with the increased risk of ovarian cancer at an adjusted OR of 2.40 (95% CI = 1.30–4.44, *P*=0.005). Similar results were found in *XPC* gene, patients carrying rs2228001 CC/AC variant genotype had higher risk of ovarian cancer when compared with those carrying AA variant genotype (adjusted OR = 1.72, 95% CI = 1.02–2.92, *P*=0.043). Conversely, rs2228000 TT/CT had decreased ovarian cancer risk in comparison with the carriers of CC genotype (adjusted OR = 0.49, 95% CI = 0.30–0.81, *P*=0.005). Moreover, a significant association was found between the *ERCC2* rs238406 polymorphism and increased risk of ovarian cancer under dominant genetic model (adjusted OR = 2.07, 95% CI = 1.07–4.01, *P*=0.032).

**Table 1:** Association between polymorphisms in nucleotide excision repair pathway genes and ovarian cancer risk

Gene	SNP	Allele		Case (*N*=89)			Control (*N*=356)			Adjusted OR^*^	P^*^	Adjusted OR^†^	*P*^†^	HWE
		A	B	AA	AB	BB	AA	AB	BB	(95% CI)		(95% CI)		
*ERCC1*	rs2298881	C	A	39	36	14	155	159	42	1.01 (0.61–1.66)	0.967	1.39 (0.69–2.81)	0.354	0.900
*ERCC1*	rs3212986	C	A	32	36	21	149	164	43	1.48 (0.88–2.48)	0.141	**2.40 (1.30–4.44)**	0.005	0.836
*ERCC1*	rs11615	G	A	63	21	5	180	147	29	**0.35 (0.20–0.61)**	**0.0002**	0.40 (0.12–1.36)	0.143	0.895
*XPA*	rs1800975	T	C	22	45	22	108	165	83	1.40 (0.79–2.47)	0.248	1.08 (0.61–1.92)	0.798	0.197
*XPA*	rs3176752	G	T	65	21	3	262	83	11	1.21 (0.71–2.08)	0.485	1.27 (0.34–4.69)	0.723	0.170
*XPC*	rs2228001	A	C	30	45	14	154	161	41	**1.72 (1.02–2.92)**	**0.043**	1.48 (0.74–2.93)	0.265	0.912
*XPC*	rs2228000	C	T	46	35	8	127	175	54	**0.49 (0.30–0.81)**	**0.005**	0.44 (0.18–1.08)	0.072	0.620
*XPC*	rs2607775	C	G	84	4	1	326	29	1	0.62 (0.21–1.81)	0.377	5.59 (0.34–90.85)	0.227	0.679
ERCC2	rs3810366	G	C	26	43	20	106	166	84	0.94 (0.55–1.61)	0.824	0.90 (0.50–1.64)	0.735	0.228
ERCC2	rs238406	G	T	13	44	32	95	168	93	**2.07 (1.07–4.01)**	**0.032**	1.60 (0.95–2.71)	0.077	0.289
ERCC2	rs13181	T	G	74	15	0	296	59	1	0.81 (0.40–1.62)	0.547	/	/	0.275
ERCC4	rs2276466	C	G	53	28	6	229	109	18	1.36 (0.82–2.27)	0.234	1.70 (0.65–4.48)	0.282	0.290
*XPG*	rs2094258	C	T	37	40	12	161	152	43	1.16 (0.70–1.91)	0.568	1.38 (0.69–2.78)	0.368	0.443
*XPG*	rs751402	C	T	34	42	13	138	167	51	0.94 (0.57–1.56)	0.811	1.17 (0.60–2.28)	0.651	0.967
*XPG*	rs2296147	T	C	58	25	6	224	122	10	0.89 (0.53–1.49)	0.658	2.18 (0.72–6.61)	0.168	0.167
*XPG*	rs1047768	T	C	49	35	5	178	149	29	0.73 (0.45–1.21)	0.220	0.61 (0.21–1.79)	0.365	0.779
*XPG*	rs873601	G	A	20	48	21	105	169	82	1.53 (0.85–2.75)	0.156	1.10 (0.62–1.95)	0.753	0.379

Abbreviations: CI, confidence interval; HWE, Hardy–Weinberg equilibrium; OR, odds ratio.

* Adjusted for age for dominant model.

† Adjusted for age for recessive model.

## Discussion

Ovarian cancer, one of the most common gynecological malignancies, is acknowledged as the fifth leading cause of cancer among female, accounting for 6% women deaths in 2011 [21]. Although a large number of investigations have tried to uncover the underlying pathogenesis of ovarian cancer, it is still difficult to make a breakthrough for the lack of tumor progression model [22]. Thus, we wanted to explore the potential biomarkers for diagnosing and predicting ovarian cancer from the angle of molecular epidemiology. Genomic integrity and stability depend on different DNA repair mechanisms, of which NER is the most flexible mechanism involving removal of various lesions, including UV-induced mutation, bulky base adducts, oxidative, and alkyl damage [23,24]. It is well known that three rare syndromes including xeroderma pigmentosum, Cockayne syndrome (CS), and the photosensitive form of the brittle hair disorder trichothiodystrophy (TTD) were induced by the deficiency of some important proteins in NER pathway [25,26]. Based on previous studies, genetic variants of the regulatory genes in NER pathway are probably related to genomic instability and even carcinogenesis.

Several investigations have evaluated the association between SNPs in NER pathway genes and prognostic of ovarian cancer [27–29]; however, a limited of research analyzed the association between the SNPs included in the present study and ovarian cancer susceptibility through retrospective case–control method. For example, Jo et al. [30] concluded that no association was found between *ERCC1* rs11615 and ovarian cancer risk in Korean women. Moreover, another study investigated the impact of two SNPs in *ERCC1* on ovarian cancer susceptibility in Chinese population, and also found that *ERCC1* rs3212986 and rs11615 polymorphisms did not show significant association with ovarian cancer risk [31] The study by Ma et al*.* [32] also concluded that there was no relationship between the *ERCC1* rs11615 and ovarian cancer. Instead, we found *ERCC1* rs11615 G>A polymorphism was associated with significantly reduced risk for ovarian cancer.

In the present study, we performed this genetic association for ovarian cancer susceptibility by genotyping 17 SNPs of six NER pathway genes in 89 patients and 356 controls. As a result, we found that both variant genotypes of *XPC* rs2228001 A>C and *ERCC2* rs238406 G>T as well as *ERCC1* rs3212986 C>A had a significant association with the increased risk of ovarian cancer under dominant and recessive genetic model respectively. On the contrary, *ERCC1* rs11615 G>A and *XPC* rs2228000 C>T polymorphisms were associated with significantly reduced risk for ovarian cancer under dominant model. To our knowledge, it is the first time to explore the association of all core genes in NER pathway with ovarian cancer.

Nonetheless, several inherent limitations of the present research still should be presented to discussion. First and obviously, the sample size in present case–control study was insufficient, which might contribute to selection bias and even decreased or increased-risk assessment. Second, many confounders influencing ovarian cancer susceptibility, such as gene–gene interaction, gene–environment interaction, and specific tumor pathologic classification, were not taken into consideration for the lack of individual information. Third, the objects in the present study were only limited in Eastern Chinese Han population, besides, other similar genetic investigations including ethnicity were not available for further integrating analysis and verifying our results. Fourth, only six genes with 17 SNPs involving in NER pathway were incorporated in this genetic association research, so other variants with potential diagnostic capability for ovarian cancer might be neglected in our research. Moreover, we were not able to measure the mRNA expression of *ERCC1, XPC*, and *ERCC2* to validate our finds because of the lack of clinic tissues.

In conclusion, our study indicated that the *ERCC1, XPC* and *ERCC2* might correlate to ovarian cancer susceptibility. However, more comprehensive studies with larger independent cohorts should be performed to unveil the real relationship between these significant genetic variations in the *ERCC1, XPC* and *ERCC2* and ovarian cancer risk.

## Supporting information

**Supplemental Table S1:** Potential functional polymorphisms in nucleotide excision repair pathway genes as predicted by SNPinfo online software

## Abbreviations

CI: confidence interval
CS: cockayne syndrome
ERCC1: excision repair cross-complementation group 1
ERCC2: excision repair cross-complementation group 2
ERCC4: excision repair cross-complementation group 4
HHR23B: human homolog B of Rad23
HWE: Hardy–Weinberg equilibrium
NER: nucleotide excision repair
OR: odds ratio
SNP: single nucleotide polymorphism
TFIIH: transcription factor II H
TTD: trichothiodystrophy
WMU: Wenzhou Medical University
XPA: xeroderma pigmentosum complementation group A
XPC: xeroderma pigmentosum complementation group C
XPG: xeroderma pigmentosum complementation group G

## References

[1] Torre L.A., Bray F., Siegel R.L., Ferlay J., Lortet-Tieulent J. and Jemal A. (2015) Global cancer statistics, 2012. CA Cancer J. Clin. 65, 87–108 10.3322/caac.21262 25651787

[2] Modugno F. and. (2003) Ovarian cancer and high-risk women-implications for prevention, screening, and early detection. Gynecol. Oncol. 91, 15–31 10.1016/S0090-8258(03)00254-3 14529658

[3] Ozols R.F., Bundy B.N., Greer B.E. (2003) Phase III trial of carboplatin and paclitaxel compared with cisplatin and paclitaxel in patients with optimally resected stage III ovarian cancer: a Gynecologic Oncology Group study. J. Clin. Oncol. 21, 3194–3200 10.1200/JCO.2003.02.153 12860964

[4] Nunns D., Symonds P. and Ireland D. (2000) Surgical management of advanced ovarian cancer. Obstet. Gynecol. Surv. 55, 746–751 10.1097/00006254-200012000-00005 11128911

[5] Ozols R.F. (2006) Challenges for chemotherapy in ovarian cancer. Ann. Oncol. 17, v181–v187 10.1093/annonc/mdj978 16807453

[6] Buschta-Hedayat N., Buterin T., Hess M.T., Missura M. and Naegeli H. (1999) Recognition of nonhybridizing base pairs during nucleotide excision repair of DNA. Proc. Natl. Acad. Sci. U.S.A. 96, 6090–6095 10.1073/pnas.96.11.6090 10339546 PMC26840

[7] Leibeling D., Laspe P. and Emmert S. (2006) Nucleotide excision repair and cancer. J. Mol. Histol. 37, 225–238 10.1007/s10735-006-9041-x 16855787

[8] de Laat W.L., Jaspers N.G. and Hoeijmakers J.H. (1999) Molecular mechanism of nucleotide excision repair. Genes Dev. 13, 768–785 10.1101/gad.13.7.768 10197977

[9] Lindahl T., Karran P. and Wood R.D. (1997) DNA excision repair pathways. Curr. Opin. Genet. Dev. 7, 158–169 10.1016/S0959-437X(97)80124-4 9115419

[10] He J., Wang F., Zhu J. (2016) Association of potentially functional variants in the XPG gene with neuroblastoma risk in a Chinese population. J. Cell. Mol. Med. 20, 1481–1490 10.1111/jcmm.12836 27019310 PMC4956948

[11] Wang M., Li Q., Gu C. (2017) Polymorphisms in nucleotide excision repair genes and risk of primary prostate cancer in Chinese Han populations. Oncotarget 8, 24362–24371 27974699 10.18632/oncotarget.13848 PMC5421853

[12] He J., Qiu L.X., Wang M.Y. (2012) Polymorphisms in the XPG gene and risk of gastric cancer in Chinese populations. Hum. Genet. 131, 1235–1244 10.1007/s00439-012-1152-8 22371296

[13] Hoeijmakers J.H. (2001) Genome maintenance mechanisms for preventing cancer. Nature 411, 366–374 10.1038/35077232 11357144

[14] Lindahl T. and Wood R.D. (1999) Quality control by DNA repair. Science 286, 1897–1905 10.1126/science.286.5446.1897 10583946

[15] Mu D. and Sancar A. (1997) Model for XPC-independent transcription-coupled repair of pyrimidine dimers in humans. J. Biol. Chem. 272, 7570–7573 10.1074/jbc.272.12.7570 9065408

[16] Wood R.D., Araujo S.J., Ariza R.R. (2000) DNA damage recognition and nucleotide excision repair in mammalian cells. Cold Spring Harb. Symp. Quant. Biol. 65, 173–182 10.1101/sqb.2000.65.173 12760031

[17] Schaeffer L., Moncollin V., Roy R. (1994) The ERCC2/DNA repair protein is associated with the class II BTF2/TFIIH transcription factor. EMBO J. 13, 2388–2392 8194528 10.1002/j.1460-2075.1994.tb06522.x PMC395103

[18] Schaeffer L., Roy R., Humbert S. (1993) DNA repair helicase: a component of BTF2 (TFIIH) basic transcription factor. Science 260, 58–63 10.1126/science.8465201 8465201

[19] O’Donovan A., Davies A.A., Moggs J.G., West S.C. and Wood R.D. (1994) XPG endonuclease makes the 3′ incision in human DNA nucleotide excision repair. Nature 371, 432–435 10.1038/371432a0 8090225

[20] Sijbers A.M., de Laat W.L., Ariza R.R. (1996) Xeroderma pigmentosum group F caused by a defect in a structure-specific DNA repair endonuclease. Cell 86, 811–822 10.1016/S0092-8674(00)80155-5 8797827

[21] Siegel R., Ward E., Brawley O. and Jemal A. (2011) Cancer statistics, 2011: the impact of eliminating socioeconomic and racial disparities on premature cancer deaths. CA Cancer J. Clin. 61, 212–236 10.3322/caac.20121 21685461

[22] Shih Ie M. and Kurman R.J. (2004) Ovarian tumorigenesis: a proposed model based on morphological and molecular genetic analysis. Am. J. Pathol. 164, 1511–1518 10.1016/S0002-9440(10)63708-X 15111296 PMC1615664

[23] Friedberg E.C. (2001) How nucleotide excision repair protects against cancer. Nat. Rev. Cancer 1, 22–33 10.1038/35094000 11900249

[24] Spivak G. (2015) Nucleotide excision repair in humans. DNA Repair 36, 13–18 10.1016/j.dnarep.2015.09.003 26388429 PMC4688078

[25] De B.J. and Hoeijmakers J.H. (2000) Nucleotide excision repair and human syndromes. Carcinogenesis 21, 453 10.1093/carcin/21.3.453 10688865

[26] Lehmann A.R. (2003) DNA repair-deficient diseases, xeroderma pigmentosum, Cockayne syndrome and trichothiodystrophy. Biochimie 85, 1101–1111 10.1016/j.biochi.2003.09.010 14726016

[27] Tang N., Lyu D., Zhang Y. and Liu H. (2017) Association between the ERCC1 polymorphism and platinum-based chemotherapy effectiveness in ovarian cancer: a meta-analysis. BMC Womens Health 17, 43 10.1186/s12905-017-0393-z 28623887 PMC5474010

[28] Fleming N.D., Agadjanian H., Nassanian H. (2012) Xeroderma pigmentosum complementation group C single-nucleotide polymorphisms in the nucleotide excision repair pathway correlate with prolonged progression-free survival in advanced ovarian cancer. Cancer 118, 689–697 10.1002/cncr.26329 21751198

[29] Deloia J.A., Bhagwat N.R., Darcy K.M. (2012) Comparison of ERCC1/XPF genetic variation, mRNA and protein levels in women with advanced stage ovarian cancer treated with intraperitoneal platinum. Gynecol. Oncol. 126, 448–454 10.1016/j.ygyno.2012.05.006 22609620 PMC3518863

[30] Jo H., Kang S., Kim S.I. (2007) The C19007T polymorphism of ERCC1 and its correlation with the risk of epithelial ovarian and endometrial cancer in Korean women. A case control study. Gynecol. Obstet. Invest. 64, 84–88 10.1159/000100008 17314486

[31] He S.Y., Xu L., Niu G., Ke P.Q., Feng M.M. and Shen H.W. (2012) Predictive value of excision repair cross-complementing rodent repair deficiency complementation group 1 and ovarian cancer risk. Asian Pac. J. Cancer Prev. 13, 1799–1802 10.7314/APJCP.2012.13.5.1799 22901125

[32] Ma Y.J., Feng S.C., Hu S.L., Zhuang S.H. and Fu G.H. (2014) Association of Rs11615 (C>T) in the excision repair cross-complementing group 1 gene with ovarian but not gynecological cancer susceptibility: a meta-analysis. Asian Pac. J. Cancer Prev. 15, 6071–6074 10.7314/APJCP.2014.15.15.6071 25124575

