# Supplementary material for: The association of polymorphisms in nucleotide excision repair genes with ovarian cancer susceptibility
Source: Biosci Rep. 2018 Jun 21;38(3):BSR20180114. doi: 10.1042/BSR20180114 (PMC6013708; doi:10.1042/BSR20180114)
Supplement: Supplementary file 1 [file bsr20180114_Supp1.pdf]

Supplemental Table 1. Potential functional polymorphisms in nucleotide excision repair pathway genes as predicted by SNPinfo online software

rs	Chr	Allele	Position	TFBS	Splicing (ESE or ESS)	Splicing (abolish domain)	miRNA (miRanda)	miRNA (Sanger)	nsSNP	Polyphen	Reg Potential	Conservation	Nearby Gene	Distance (bp)	Allele	Asian	CHB
rs2298881	19	A/C	50618756	Y	--	--	--	--	--	--	0.252611	0	ERCC1	14044||261	C	0.627	0.661
rs3212986	19	C/A	50604576	--	--	--	--	--	Y	benign	0.305187	0	CD3EAP	3269||1288	C	0.617	0.685
rs11615	19	G/A	50615493	--	Y	--	--	--	--	--	0.26724	0.989	ERCC1	10781||3524	G	0.637	0.667
rs1800975	9	T/C	99499399	Y	Y	--	--	--	--	--	0.483269	0	XPA	22387||113	C	0.592	0.644
rs3176752	9	T/G	99477308	--	--	--	Y	Y	--	--	0.112079	0	XPA	296||22204	G	0.583	0.625
rs2228001	3	T/G	14162450	--	--	--	--	--	Y	--	0.189938	1	XPC	802||32693	T	0.610	0.685
rs2228000	3	G/A	14174889	--	--	--	--	--	Y	--	0.136701	0	XPC	13241||20254	G	0.617	0.685
rs2607775	3	C/G	14195099	Y	Y	--	--	--	--	--	0.282058	0	XPC	33451||44	C	0.624	0.696
rs3810366	19	C/G	50565782	Y	--	--	--	--	--	--	0.174384	0	ERCC2||PPP1R13L	-113||-8954	C	0.611	0.685
rs238406	19	G/T	50560149	--	Y	Y	--	--	--	--	0.36557	0.996	ERCC2	13463||5520	G	0.843	0.768
rs13181	19	G/T	50546759	--	Y	Y	--	--	Y	benign	0.585468	0.999	ERCC2	73||18910	T	0.858	0.791
rs2276466	16	C/G	13950676	--	--	--	Y	Y	--	--	0.174235	0	ERCC4||MKL2	-971||-122021	C	0.838	0.768
rs2094258	13	T/C	102294760	Y	--	--	--	--	--	--	0	0.001	BIVM||ERCC5	-2878||-1415	C	0.833	0.768
rs751402	13	G/A	102296199	Y	Y	Y	--	--	--	--	0.25613	0	ERCC5	24||30147	G	0.838	0.789
rs2296147	13	C/T	102296376	Y	--	--	--	--	--	--	0.175993	0	ERCC5	201||29970	T	0.856	0.768
rs1047768	13	C/T	102302518	--	Y	Y	--	--	--	--	0.24405	0.914	ERCC5	6343||23828	T	0.844	0.774
rs873601	13	A/G	102326338	--	Y	Y	Y	Y	--	--	0	0.005	ERCC5	30163||8	G	0.874	0.807
